# COVID-19 and Dentistry in 72 Questions: An Overview of the Literature

**DOI:** 10.3390/jcm10040779

**Published:** 2021-02-16

**Authors:** Stéphane Derruau, Jérôme Bouchet, Ali Nassif, Alexandre Baudet, Kazutoyo Yasukawa, Sandrine Lorimier, Isabelle Prêcheur, Agnès Bloch-Zupan, Bernard Pellat, Hélène Chardin, Sophie Jung, on behalf of TASK FORCE COVID-19–Collège National des EnseignantS en Biologie Orale (CNESBO)—France

**Affiliations:** 1 UFR Odontologie, Université de Reims Champagne-Ardenne, 51100 Reims, France; stephane.derruau@univ-reims.fr (S.D.); sandrine.lorimier@univ-reims.fr (S.L.); 2 Pôle de Médecine Bucco-dentaire, Centre Hospitalier Universitaire de Reims, 51092 Reims, France; 3 BioSpecT EA-7506, UFR de Pharmacie, Université de Reims Champagne-Ardenne, 51096 Reims, France; 4 UFR Odontologie-Montrouge, Université de Paris, 92120 Montrouge, France; jerome.bouchet1@u-paris.fr (J.B.); bernard.pellat@u-paris.fr (B.P.); helene.chardin@u-paris.fr (H.C.); 5 Laboratory “Orofacial Pathologies, Imaging and Biotherapies” URP 2496, University of Paris, 92120 Montrouge, France; 6 UFR Odontologie-Garancière, Université de Paris, 75006 Paris, France; alinassifodf@gmail.com; 7 AP-HP, Sites hospitaliers Pitié Salpêtrière et Rothschild, Service d’Orthopédie Dento-Faciale, Centre de Référence Maladies Rares Orales et Dentaires (O-Rares), 75013-75019 Paris, France; 8 INSERM, UMR_S 1138, Laboratoire de Physiopathologie Orale et Moléculaire, Centre de Recherche des Cordeliers, 75006 Paris, France; 9 Faculté de Chirurgie Dentaire, Université de Lorraine, 54505 Vandœuvre-lès-Nancy, France; alexandre.baudet@univ-lorraine.fr (A.B.); kazutoyo.yasukawa@univ-lorraine.fr (K.Y.); 10 Centre Hospitalier Régional Universitaire de Nancy, 54000 Nancy, France; 11 Université de Reims Champagne-Ardenne, MATIM EA, UFR Sciences, 51687 Reims, France; 12 Faculté de Chirurgie Dentaire, Université Côte d’Azur, 06000 Nice, France; isabelle.precheur@univ-cotedazur.fr; 13 Pôle Odontologie, Centre Hospitalier Universitaire de Nice, 06000 Nice, France; 14 Laboratoire Microbiologie Orale, Immunothérapie et Santé (MICORALIS EA 7354), Faculté de Chirurgie Dentaire, 06300 Nice, France; 15 Faculté de Chirurgie Dentaire, Université de Strasbourg, 67000 Strasbourg, France; agnes.bloch-zupan@unistra.fr; 16 Pôle de Médecine et de Chirurgie Bucco-Dentaires, Centre de Référence Maladies Rares Orales et Dentaires (O-Rares), Hôpitaux Universitaires de Strasbourg, 67000 Strasbourg, France; 17 Institut de Génétique et de Biologie Moléculaire et Cellulaire (IGBMC), INSERM U 1258, CNRS UMR 7104, Université de Strasbourg, 67400 Illkirch-Graffenstaden, France; 18 AP-HP, Hôpital Henri Mondor, 94010 Créteil, France; 19 ESPCI, UMR CBI 8231, 75005 Paris, France; 20 INSERM UMR_S 1109 «Molecular Immuno-Rheumatology», Institut Thématique Interdisciplinaire de Médecine de Précision de Strasbourg, Transplantex NG, Fédération hospitalo-universitaire OMICARE, Fédération de Médecine Translationnelle de Strasbourg, Université de Strasbourg, 67000 Strasbourg, France

**Keywords:** COVID-19, dental practice, dentistry, oral health, SARS-CoV-2

## Abstract

The outbreak of Coronavirus Disease 2019 (COVID-19), caused by Severe Acute Respiratory Syndrome Coronavirus 2 (SARS-CoV-2), has significantly affected the dental care sector. Dental professionals are at high risk of being infected, and therefore transmitting SARS-CoV-2, due to the nature of their profession, with close proximity to the patient’s oropharyngeal and nasal regions and the use of aerosol-generating procedures. The aim of this article is to provide an update on different issues regarding SARS-CoV-2 and COVID-19 that may be relevant for dentists. Members of the French National College of Oral Biology Lecturers (“Collège National des EnseignantS en Biologie Orale”; CNESBO-COVID19 Task Force) answered seventy-two questions related to various topics, including epidemiology, virology, immunology, diagnosis and testing, SARS-CoV-2 transmission and oral cavity, COVID-19 clinical presentation, current treatment options, vaccine strategies, as well as infection prevention and control in dental practice. The questions were selected based on their relevance for dental practitioners. Authors independently extracted and gathered scientific data related to COVID-19, SARS-CoV-2 and the specific topics using scientific databases. With this review, the dental practitioners will have a general overview of the COVID-19 pandemic and its impact on their practice.

## 1. Introduction

Severe Acute Respiratory Syndrome Coronavirus 2 (SARS-CoV-2) is the cause of the current Coronavirus Disease 2019 (COVID-19) pandemic, whose first case was reported in December 2019 in Wuhan, Hubei province, China. In January 2021, the pandemic is still ongoing and is getting worse [1]. Dental surgery is considered to be a profession at high risk for being infected, and therefore transmitting SARS-CoV-2. Our professional practice was disrupted by lockdowns, resulting in reduced activity, new dental protocols and additional costs for staff protective equipment. This has caused unexpected financial difficulties for many dental practitioners. Even with treatments or vaccines, our professional practice will probably never revert back to the previous situation, as the new constraints may become permanent.

The aim of this article is to provide an update on issues dentists may encounter with SARS-CoV-2/COVID-19 or that are not addressed in recommendations to dental professionals.

To compose this integrative review, a panel of questions susceptible to be of major interest for the dental community has been selected. The questions were selected after discussion between the members of the working group, which is mostly composed of dentists and experienced dental researchers that are members of the French National College of Oral Biology Lecturers (“Collège National des EnseignantS en Biologie Orale”; CNESBO-COVID19 Task Force). Questions were grouped in 10 different major topics that made up the different sections of the manuscript. 

To answer these questions, a wide range of keywords was chosen to cover all the topics that are discussed. In total, 378 references were selected in this review. Original studies and significant reviews were included, based on their importance regarding the chosen topics, but also websites from relevant national and international health agencies (e.g., World Health Organization (WHO), Center for Disease Control and Prevention (CDC)). The time period covered by this review gathers published literature from the onset of the COVID-19 pandemic until mid-January 2021.

Q1—What is the impact of COVID-19 on dental practice?

In the Hospital of Stomatology from Wuhan, nine dental staff members and students were infected from 23 January to 4 February 2020 [2]. Chinese dental surgeons immediately responded with recommendations for the management of patients in the context of the epidemic [2,3]. Since then, recommendations have been published on professional websites in many countries, for example in the US (Centers for Disease Control and Prevention (CDC), American Dental Association), in Europe (European Centre for Disease Prevention and Control (ECDC)), in the UK (National Health Service, British Dental Association), in France (Health Ministry, French Dental Association). During the first epidemic wave, the most affected countries put in place a general lockdown, with the closure of dental offices. Only dental emergency services and teleconsultations were authorized. Then, dental offices reopened, with strict conditions for sorting and receiving patients, and detailed protocols for staff protection and to carry out dental care. These recommendations are still ongoing [4,5]. The economic impact is worrying. Besides, fear of contracting and transmitting the virus has caused work-related stress, and sometimes premature retirement of dental surgeons [6,7,8] 

## 2. Worldwide COVID-19 Epidemiology

Q2—What was the starting point of the pandemic?

At the end of 2019, several cases of “pneumonia of unknown cause” were identified in Wuhan, and a new coronavirus, SARS-CoV-2, was rapidly identified [9,10]. An outbreak of zoonotic origin was suspected, as bats are the natural reservoir of many coronaviruses. Transmission to humans may be mediated by intermediate animals [11]. Attention was focused on the Wuhan wholesale market, which trades in a variety of live animals, but not bats. Genomic analysis confirmed that SARS-CoV-2 shared 96.2% identity with a bat coronavirus (BatCoV RaTG13), and 91.02% identity with a Pangolin-CoV, newly identified from Wuhan market [12]. Direct contact with pangolins, or meat consumption, were suspected to be the main source of transmission of SARS-CoV-2 [13]. However, in the initial cohort of 41 hospitalized patients, 14 patients had no direct exposure to Wuhan market [14]. In particular, the first patient identified had no reported connection with the Wuhan market, or with subsequent cases. His respiratory symptoms began on 1 December 2019, indicating that SARS-CoV-2 was circulating in Wuhan in November 2019. The 7th edition of the World Military Summer Games, which took place in Wuhan and ended October 27, is suspected to have been an early cluster. To date, the starting point of COVID-19 pandemic is still unknown [15,16]. 

Q3—Why did the initial outbreak turn into a pandemic?

The COVID-19 outbreak arose at the time of the Chinese New Year holidays with large movements of travelers across China. Holidays began on 21 January 2020. Chinese Authorities quarantined Wuhan on January 24 and implemented severe control measures [1,17,18]. In early 2020, Health Authorities from various countries estimated that they could stop COVID-19 by applying the same control measures as for SARS (Severe Acute Respiratory Syndrome) pandemic (2002–2003) and MERS (Middle East Respiratory Syndrome) pandemic (2012, still ongoing). However, scientific studies have progressively shown that SARS-CoV-2 was more contagious than SARS-CoV and MERS-CoV [19]. SARS-CoV-2 is easily transmitted by droplets from person to person, and via contaminated surfaces. Asymptomatic people may be contagious, and sick people are contagious before, during and after clinical symptoms onset [20]. As a result, temperature checking was not sufficient to detect virus carriers. Travelers arriving from Wuhan before January 24 were able to transmit SARS-CoV-2 throughout China and then to Thailand and other countries. In addition, on January 30, the World Health Organization (WHO) “believed that it was still possible to stop virus spread by applying strong preventive measures at the international level,” but did not ban travel and trade [21]. Travel controls and preventive measures have been gradually introduced by various countries, but too late [22]. 

Q4—What is the extent of the pandemic today?

COVID-19 epidemiologic data vary according to sources, such as Johns Hopkins University (JHU) coronavirus resource center or WHO situation updates. Mid-January 2021, global data approached 91 million cases and 2 million deaths worldwide [23]. According to JHU [1], current global mortality rate of COVID-19 is 2.2%. As a comparison, the mortality rate of SARS was 9.6%, MERS was 34.5% and pandemic flu H1N1 (2009–2018; pdm09 virus) were 0.07% [24]. There are major differences between countries that depend on geographical and demographic factors, and on the political will to communicate the data transparently. Infection fatality rate for COVID-19 is below 1% under 50 years, with an exponential increase over 60 years, ranging from 2.5% in the age group 65–74 years, to around 28% over 80 years [25]. According to JHU reports, there was an initial epidemic peak in China on 13 February 2020, followed by three pandemic waves worldwide in April–May, August–September and November–December-January (still ongoing) [1]. A fourth wave has been described in Hong Kong. Vaccination began in some countries in December 2020, but at the beginning of January 2021, its impact is not yet noticeable. Taking into account the number of cases, the ten most affected countries are currently the US (>23 million cases, >388,000 deaths), followed by India, Brazil, Russia, United Kingdom, France, Turkey, Italy, Spain, and Germany [1]. 

Q5—What is the effectiveness of preventive measures implemented?

Preventive measures aim at slowing down the transmission of the virus via social distancing, face masks, hand hygiene, avoidance of crowds and poorly ventilated spaces, contact tracing, rapid testing and isolation [26]. At the beginning of the pandemic, many countries attempted to detect and quarantine at-risk travelers, identify clusters and isolate confirmed patients. This strategy was not efficient, and lockdown was imposed [26]. The aim was to “flatten the curve” of new contaminations, and to avoid the saturation of hospitals and intensive care units. Teleworking, banning cultural, sports, and family gatherings, closure of schools, universities, non-essential businesses have had a heavy psychological and economic impact. In China, lockdown and all preventive measures have been applied with highest severity. It was accepted by the population, which has made it possible to stop the virus transmission [17]. In addition, protective equipment is mostly manufactured in China [17]. Initially, in some countries, medical teams and populations could not be properly equipped [8]. In January 2021, the pandemic seems under control in China and in some other countries [1]. Elsewhere, preventive measures were implemented too late, insufficient or poorly accepted because all nations do not share the same idea of civil liberties [17]. The pandemic continues to spread rapidly [26,27]. 

Q6—Is there a risk to be co-infected with SARS-CoV-2 and other respiratory pathogens?

As with other acute respiratory infections, microbial superinfection is common in people infected with SARS-CoV-2 [19]. In a series of 257 subjects, 94.2% of cases had co-infection, and 9 viruses, 11 bacteria and 4 fungi were detected. The most common were bacterial superinfections due to *Streptococcus pneumoniae*, *Klebsiella pneumoniae* and *Haemophilus influenzae*. The other germs most often isolated were a fungus (*Aspergillus*) and a virus (Epstein Barr Virus; EBV). At a lower rate, other bacteria (*Escherichia coli, Staphylococcus aureus, Pseudomonas aeruginosa*), viruses (Rhinovirus, Adenovirus, Herpes virus, but rarely Influenza virus A or B) and fungi (*Mucor, Candida spp.*) were detected [28]. In a series of 2188 patients, respiratory viruses were identified, mostly Bocavirus, followed by Respiratory Syncytial and Parainfluenza viruses [29]. However, the boundaries between viral/viral co-colonization, superinfection or successive infections must be clarified. The diagnosis of bacterial or fungal superinfections is easier. Overall, co-infections aggravate respiratory signs and the risk of severe or critical COVID-19 by weakening the immune system (see Q20). There is no association between SARS-CoV-2 and specific respiratory pathogens, but influenza vaccine appears more than ever to be recommended for dental surgeons, in order to avoid two successive acute respiratory infections [28,30]. 

## 3. SARS-CoV-2 Virology

Q7—Where does the virus come from? Are there some other pathogenic coronaviruses?

Human coronaviruses, discovered in the 1960s, are part of the Coronaviridae family and the Nidovirales order [31]. These are enveloped viruses with unsegmented, single-stranded RNA of positive polarity approaching 30,000 nucleotides (Baltimore Classification Group IV [32,33]). Among the Coronaviridae, 7 strains of coronavirus are known to infect humans. Four are considered to be responsible for benign respiratory infections such as the “common colds” (HCoV-229E, -OC43, -NL63 and -HKU1) and three strains, identified more recently, can cause the development of serious, potentially fatal pneumopathies. SARS-CoV and MERS-CoV were discovered in 2002 and 2012, respectively, while SARS-CoV-2, named because of its similarity to SARS-CoV, was discovered in 2019 [34,35].

Q8—What is SARS-CoV-2 as a virus?

Coronaviruses are enveloped viruses characterized by the presence of spikes (S) made up of glycoproteins, found in trimeric form and embedded in the viral envelope. These spikes, arranged in the shape of a crown around the viral membrane, give their name to the coronaviruses. The genomic RNA (gRNA) is encapsulated in a nucleocapsid (N) of helical shape. The whole genomic RNA and the nucleocapsid (N), called ribonucleoprotein (RNP), are enveloped in the viral particle using membrane (M) and envelope (E) glycoproteins [36]. The SARS-CoV-2 genome enables the transcription of gRNA as well as of 9 major subgenomic RNAs [37]. From the complete genomic RNA, two polypeptides are translated according to their open reading frame. Their autocleavage allows the release of about 26 non-structural proteins essential for virus replication, among which are the proteins of the replicase-transcriptase complex [37]. Subgenomic RNAs allow the expression of structural proteins (N, M, E and S) common to all coronaviruses, and of certain non-structural and accessory proteins, which are all virulence factors [36,37].

Q9—How does the virus penetrate cells?

The spike (S) surface protein interacts through its receptor-binding domain (RBD) with the cell surface receptor ACE2 [38]. ACE2 is the angiotensin 2 converting enzyme, whose function is to decrease the plasma concentration of angiotensin, thereby causing vasoconstriction and regulation of blood pressure [39]. This receptor is common to several strains of coronavirus, including SARS-CoV, SARS-CoV-2 and HCoV-NL63 [38,40,41]. After SARS-CoV endocytosis, an interaction of the viral protein S with the transmembrane serine 2 protease (TMPRSS2) mediates its cleavage [42,43], thus exposing the fusogenic peptide of protein S and allowing subsequent fusion between the viral envelope and the membrane of endocytosis vesicles [38,40,44]. 

Q10—How does the virus replicate?

After entering the cell cytosol, viral genomic RNA, which is 3’polyadenylated, is directly translated by cellular ribosomes into non-structural polypeptides which are self-cleaved by their proteolytic activity and reassembled into a RNA-dependent replicase protein complex [45]. This allows RNA replication into genomic RNA or subgenomic RNAs. The subgenomic RNAs are then translated into structural proteins (N, M, E and S) and accessory proteins, which assemble into new virions at the level of an intermediate compartment between the endoplasmic reticulum and Golgi apparatus [46,47]. The fusion of the vesicles containing the viral particles with the cell plasma membrane allows the release by exocytosis of the virions into the extracellular medium [45]. 

Q11—Which cells/organs are infected by SARS-CoV-2 and how does SARS-CoV-2 spread in infected organism?

As SARS-CoV [48], SARS-CoV-2 is a multiple organ targeting virus. The abundant epithelial expression of ACE2 (angiotensin 2 converting enzyme) is thought to provide a route for virus entry into the organism, while its vascular endothelial expression may help the virus replication and spreading within the organism [49]. The importance of host proteases, mainly TMPRSS2 (transmembrane serine 2 protease), in SARS-CoV-2 entry has been evidenced [50]. Using single-cell RNA sequencing, Ziegler et al. identified the tissue-resident cells subsets expressing both ACE2 and TMPRSS2 proteins. They found that secretory goblet cells, type II pneumocytes and absorptive enterocytes were the primary targets of SARS-CoV-2, thus explaining the high replication rate of the virus in these tissues, and the associated symptoms [51]. Finally, as glial cells and neurons express ACE2, they have been suspected of being targets for SARS-CoV-2 infection [52,53], in agreement with the neurological manifestations observed in a large proportion of COVID-19 patients [54].

Q12—Does the virus evolve?

Thanks to the proofreading activity of their polymerase (nucleic acid repair activity), coronaviruses exhibit a lower mutation rate than other RNA viruses [55]. Nonetheless, several mutants of SARS-CoV-2 have been described [56]. Mutations on the S protein are closely monitored because they could involve some modification of the virus virulence, as well as the emergence of resistance against vaccines targeting this protein. Very early in the development of the epidemic, a D614G mutation (aspartic acid into glycine) was described as increasing infectivity. This mutation presented a selection advantage, as this subtype of SARS-CoV-2 is now the major variant worldwide [57]. More recently, a set of new mutations in the spike (S) protein (viral strain B.1.1.7) has been described in the UK, as probable evolutionary advantages for the virus, increasing its dissemination ability [58]. 

## 4. Immunology of COVID-19

Q13—What are the main characteristics of the innate immune response against SARS-CoV-2?

The efficacy of the innate immunity against viral infections relies on the early and robust type I interferon (IFN) responses, which promotes viral clearance and induction of adequate adaptive immunity [59,60]. SARS-CoV-2 is able to evade immune system recognition, to suppress the activation of the innate immune system, and to dampen type I IFN responses [61,62,63,64,65]. This is supported by the observation that very rare genetic defects causing primary immunodeficiency of type I IFN immunity and autoantibodies against type I IFNs are more commonly found in patients with life-threatening COVID-19 [66,67]. These viral immune evasion strategies allow uncontrolled SARS-CoV-2 replication without triggering the innate anti-viral response machinery of epithelial cells [63]. However, at a later stage, infected cells undergo cell death, particularly in the airways, resulting in lung injury. The important release of viral particles triggers the production of high levels of pro-inflammatory cytokines (e.g., IL-1β, IL-6, TNF-α). Failure to control SARS-CoV-2 infection at early stages in the respiratory tract may in some cases lead to a dysregulated systemic hyperinflammation called “cytokine storm”, in a second phase of the disease (see Q18) [59].

Q14—What are the main characteristics of the adaptative immune response against SARS-CoV-2?

Adaptive immunity involves both humoral (mediated by antibodies) and cellular (mediated by T lymphocytes) responses. However, lymphopenia has been shown to be one of the most prominent markers of COVID-19 [59,68,69,70,71]. 

Humoral immunity to SARS-CoV-2 is mediated by antibodies directed against surface proteins of the virus. Antibodies are important for viral neutralization and clearance, but also play a role in the modulation of immune responses. The neutralizing antibodies mainly target the spike (S) protein (in particular the receptor-binding domain RBD), thus blocking the interaction between SARS-CoV-2 and ACE2 and inhibiting the virus entry into host cells, but also the nucleocapsid (N) protein. In most infected individuals, anti-SARS-CoV-2 IgM and IgG antibodies are detectable within 1-2 weeks (median: 11 days [72]) after symptoms onset (see Q24) [73]. IgM are typically the first produced antibodies, but some authors have found that the IgA response peaks earlier and may be more pronounced [74,75]. However, the detection of antibodies against SARS-CoV-2 does not indicate directly protective immunity and the kinetics of neutralizing antibodies is yet unclear (see Q17). A strong antibody response appeared to correlate with more severe clinical disease [76,77]. Sex differences have also been reported, with males displaying higher antibody levels shortly after infection, but a faster decrease of neutralizing antibodies at 3–6 months [78].

Regarding cellular adaptive immunity, both CD4^+^ helper T lymphocytes and CD8^+^ cytotoxic T lymphocytes are crucial for optimal antibody production and lysis of virus-infected cells [79]. They also secrete cytokines that drive the recruitment of other immune cells. SARS-CoV-2-specific CD8^+^ and CD4^+^ T-cell responses are found in most COVID-19 patients within 1–2 weeks [80,81]. Similar to other viral infections, SARS-CoV-2-specific CD4^+^ T cells predominantly possess a Th_1_ phenotype (that lead to an increased cell-mediated response) [79]. A decrease in the number of T cells has been reported in patients with more severe forms of COVID-19, suggesting that strong T-cell responses may be correlated with milder disease [59,68,69,70]. In addition, reduced functional diversity and elevated T-cell exhaustion (i.e., dysfunction with loss of effector functions) contribute to severe progression [80]. Some individuals exposed to SARS-CoV-2 develop specific T-cell memory responses (see Q17) but no specific antibodies, suggesting that cellular immunity might be induced in the absence of humoral immune responses [82,83]. 

Q15—Are there differences in the immune responses between symptomatic and asymptomatic individuals?

Approximately 45% of SARS-CoV-2 infections may be asymptomatic [84] but importantly, asymptomatic carriers have been proven to be contagious [85]. Several differences in immune responses have been observed between symptomatic and asymptomatic individuals. First, the duration of viral shedding is longer in asymptomatic individuals [86]. Second, IgG titers were reported to be significantly lower in asymptomatic individuals compared to symptomatic patients, with a faster decrease of antibody responses (40% of asymptomatic individuals become seronegative within 2–3 months versus 13% of symptomatic patients) [86]. Conversely, many individuals with asymptomatic or mild COVID-19 seem to have highly durable memory T-cell responses, even in the absence of detectable humoral responses [82]. The level of “herd immunity” (i.e., population immunity) can therefore not be extrapolated from serology studies only.

Q16—Are there differences in the immune responses between adults and children?

Children are underrepresented in the total burden of COVID-19 (about 2%; see Q37) [87]. Except rare cases of life-threatening multisystem inflammatory syndrome (MIS-C or Kawasaki-like hyperinflammatory syndrome) [88,89], children tend to develop a milder disease and a large proportion of infected children are asymptomatic (see Q37) [87,90,91], probably resulting in an under-estimation of SARS-CoV-2 infection in this population [92]. Different mechanisms have been proposed. First, the expression of ACE2 receptors in the airway epithelial cells appears to be lower in children [93,94]. Second, children may exhibit more robust innate immune responses [89,95]. They also have the ability to produce more rapidly than adults the so-called natural antibodies (IgM) that play an important role in early phases of infection as they are present prior to antigen encounter. Owing to their high reactivity, they contribute to containing the infection until specific antibodies are produced [96,97]. Third, previous infection by seasonal endemic coronaviruses, which are very frequent in children, could confer a certain degree of cross-reactive immunity to SARS-CoV-2 (see Q19) [98]. It has also been suggested that frequent vaccinations and repeated infections might result in a more “trained immunity” (i.e., form of memory exhibited by the innate immune system) [99,100]. Fourth, adaptive immune responses differ in pediatric and adult populations. In contrast with COVID-19 adult patients, which present high rates of lymphopenia [59,68], white blood cell counts are within the normal ranges in most children [90]. Both quantitative and qualitative differences have been observed in the specific antibody response. Children have a reduced breadth of anti-SARS-CoV-2 specific antibodies and a lower neutralizing activity as compared to adult COVID-19 cohorts [101]. The reduced functional antibody response could be due to a more efficient immune-mediated viral clearance [101]. Pediatric T-cell responses to SARS-CoV-2 may exceed those of adults as children present a higher number of naive T cells [102]. 

Q17—What do we know about long-term protective immunity to SARS-CoV-2 infection?

Long-term immunity relies on memory T and B lymphocytes, the latter being able to produce antibodies for a long time. Evaluating its duration and strength in the protection against reinfection is a key issue to predict the course of COVID-19 pandemic. Indeed, cases of SARS-CoV-2 reinfection have been reported [103,104,105,106,107], some resulting in worse disease outcomes than at first infection [104,105]. Insight can be gained from previous studies on other human coronaviruses [108]. Protective immunity to seasonal coronaviruses responsible for “common colds” is short-lasting with frequent reinfections [108,109]. In SARS, serum antibody titers remain elevated for the first 2 years, but then decrease significantly over time with undetectable memory B cell responses at 6 years. However, SARS-CoV specific T-cells have been shown to persist more than 10 years after infection [110,111,112,113,114]. 

Regarding SARS-CoV-2, some authors observed a decline in specific IgG and neutralizing antibodies titers after an initial peak [115]. One study revealed that 40% of asymptomatic and 13% of symptomatic infected individuals, after showing anti-SARS-CoV-2 IgG positivity, reverted back to seronegativity in the early convalescent phase [86]. In addition, antibody responses were not detectable in all patients, especially asymptomatic individuals or with mild forms of COVID-19 [86]. Other studies have however shown a relative stability of antibodies titers [116,117] for more than 6 months, with S-specific memory B cells that were more abundant at 6 months than at 1 month post symptom onset [117]. 

SARS-CoV-2-specific memory T cells have been detected in most convalescent individuals, including asymptomatic cases and those with undetectable antibody responses [80,82,118]. Remarkably, more than 90% of ‘‘exposed asymptomatic’’ individuals exhibited detectable T cell responses to SARS-CoV-2, despite 60% of them only being seropositive [82,119]. However, a recent study showed that SARS-CoV-2 specific memory T cells declined with a half-life of 3-5 months [117]. Further studies are therefore strongly needed to assess the kinetics of long-term immunity and to evaluate the efficiency of memory responses against reinfection. 

Q18—What does the expression “cytokine storm” mean?

Between 5 and 10% of COVID-19 patients may develop a severe form requiring critical care management, with a high mortality rate [59,120]. Rapidly progressing clinical deterioration is generally observed in the advanced stages of COVID-19 (7-10 days after symptoms onset), with the development of acute respiratory distress syndrome (ARDS), accompanied by a state of aggressive systemic hyperinflammation in a condition termed “cytokine storm” [121]. Notably, ARDS occurs despite a decreasing viral load, suggesting that it may be due to an exuberant host immune response, rather than to viral virulence [59]. Normal anti-viral immune responses require the activation of inflammatory pathways and the production of proinflammatory cytokines (IL-1β, IL-6, TNF-α, type I IFNs) [122]. However, in some cases, a dysfunctional immune reaction can lead to an uncontrolled release of pro-inflammatory cytokines [123]. The “cytokine storm” is not a specific complication of COVID-19 and can be associated with a variety of other infectious (e.g., influenza, SARS, MERS) and non-infectious diseases [121,124]. It produces an excessive inflammatory feedforward loop, which starts at a local site (in the lungs in COVID-19) but rapidly spreads throughout the body and drives the pathology. It is responsible for vascular hyperpermeability, coagulopathy, widespread tissue damage, leading multi-organ failure with ARDS, and ultimately death [125,126,127]. Several factors have been involved and include rapid viral replication in the early stages of infection, resulting in high proinflammatory responses. Surprisingly, SARS-CoV-2 is also able to dampen the host immune responses, inducing a state of immunodeficiency, which contributes to a less controlled inflammatory response [126] (see Q20). 

Underlying uncontrolled diseases that are characterized by an hyperinflammatory state such as diabetes, but also possibly generalized periodontitis, may increase the risk of developing severe forms of COVID-19 [128,129,130]. The presence of diabetes in patients with COVID-19 is associated with a significant increase in severity and mortality [129]. Various hypotheses have been proposed to explain this correlation, including a dental hypothesis [131], diabetes being a risk factor for periodontal diseases. Although there is currently insufficient evidence to link periodontal diseases with an increased risk of SARS-CoV-2 infection, some authors have observed a higher mortality for COVID-19 patients with periodontal diseases [130,132]. 

The development of treatments targeting the cytokine storm (i.e., anti-cytokine therapy or immunomodulators; see Q41) will be crucial for patients with severe COVID-19. However, this strategy must be balanced with the maintenance of an adequate inflammatory response for virus clearance [127].

Q19—Can previous exposure to “common cold” coronaviruses protect against SARS-CoV-2 infection?

Four strains of coronaviruses (see Q7) have been shown to be responsible for around 15% of “common colds” in humans [108]. It has been suggested that previous infection with these seasonal endemic coronaviruses could confer a certain degree of cross-reactive immunity to SARS-CoV-2 [98]. This can be explained by a relatively high amino acid similarity between recognized SARS-CoV-2 and seasonal coronaviruses epitopes [79]. Indeed, T cells reactive to SARS-CoV-2 have been detected in 20% to 60% of healthy individuals without known exposure to the virus [80,110,133]. It has been estimated that more than 90% of adults have serum antibodies specific for the common cold coronaviruses (that could potentially cross react with SARS-CoV-2 epitopes) [108], but their titers wane rapidly within months after infection, with only a weak protection against reinfection [63,98,109]. Although we still lack direct evidence that recent exposure to seasonal coronaviruses can reduce COVID-19 severity (this could also contribute to an increase in inflammatory signals [79]), understanding the protective value of pre-existing SARS-CoV-2-reactive T cells will therefore be crucial, in particular since cross-reactive immune responses can be boosted through vaccination and contribute to an increased vaccine-induced protective immunity [63].

Q20—Are patients with immunodeficiencies/under immunosuppressants at higher risk to develop severe COVID-19?

Immunodepression may be a “double-edged sword” in SARS-CoV-2 infection [134]. On the one hand, an immunocompromised state may predispose to infections and facilitate virus spreading. Patients with a compromised immune status (e.g., HIV infection, cancer, primary immunodeficiencies, history of solid organ transplantation, immunosuppressive/modulating treatments) have been identified as being at higher risk of developing severe forms of COVID-19 both in Europe (European Centre for Disease Prevention and Control; ECDC) and the US (Centers for Disease Control and Prevention; CDC) [135,136]. The risk seems even increased as SARS-CoV-2 itself induces lymphopenia [14,71], favoring the development of secondary infections (see Q6). On the other hand, in advanced stages of COVID-19, immunosuppression may be beneficial in countering immune-mediated damage due to excessive inflammation, particularly in the context of “cytokine storm” (see Q18). Several immunosuppressive therapies are currently under investigation or at various phases of development to control or prevent the development of this complication (see Q41) [59,137]. Current knowledge on the impact of immunosuppression on SARS-CoV-2 infection is still limited with varying results between studies and depending on the cause of immunosuppression [138,139,140,141,142,143,144,145,146,147,148]. Patients suffering from cancer seem to represent the highest risk subgroup [140,144,145]. Regarding COVID-19 patients with primary immunodeficiencies, more than one third presented only a mild form of COVID-19 and the risk factors predisposing to severe disease were comparable to those in the general population [148]. A higher prevalence of COVID-19 has been observed in patients with systemic autoimmune diseases, particularly in those without ongoing conventional immunosuppressants [147]. However, the risk of complications appeared to be similar when compared to the general population [146]. Patients under immunosuppressive/modulating therapy without suspected or confirmed COVID-19 should continue their treatment without modification, unless otherwise indicated by the patient’s expert physician, as recommended by national and international societies [149,150,151]. Until reliable data are available, a close clinical monitoring and social distancing should be prioritized for these patients.

Q21—What is the role played by oral/mucosal immunity in SARS-CoV-2 infection?

To date, very little is known about mucosal immune responses at the sites of SARS-CoV-2 infection. As this virus mainly penetrates mucosal epithelial cells, mucosal immunity may be an important parameter influencing the infection course. The induction of a strong local immune response may be crucial for the initial control of the virus and for paving the way to an effective adaptive immune response [152]. Mucosal immune responses are initiated at inductive sites in nasopharynx-associated lymphoid tissues and lead to the production of secretory IgA. The latter play a crucial role in the exclusion of pathogens from the upper respiratory tract mucosal surfaces. During SARS-CoV-2 infection, IgG, IgA and IgM antibodies directed against the Spike (S) protein and the receptor-binding domain (RBD) of the S protein are detectable in the saliva, but only the IgG response seems to persist beyond day 60 [153]. A better understanding of mucosal immune responses will be crucial, as they may have important implications for vaccine design, in particular for the development of mucosal immunization strategies (see Q45) [154,155]. 

Q22—Can the microbiota play a role in the course of SARS-CoV-2 infection?

The microbiota is crucial for maintaining mucosal homeostasis. Indeed, a persistent imbalance of microbial communities, named dysbiosis, can lead to dysregulated immune responses with hyperinflammation. A dysbiosis profile has been observed in COVID-19 patients, particularly in those presenting a severe form of the disease and/or with pre-existing comorbidities [156,157,158,159]. Future studies are needed to understand the interactions between the microbiome and SARS-CoV-2, and the influence of the microbiota on the course of the disease. The therapeutic potential of microbiota modulation should also be evaluated in this context.

## 5. Diagnosis and SARS-CoV-2 Detection 

Q23—What are the various tests to diagnose COVID-19?

Samples are generally obtained using nasopharyngeal swabs (NPS) but also from the oral cavity, as high viral loads are found both in the respiratory tract and the saliva [160,161] (see Q31). The highest viral loads are usually detected in the airways 5 to 6 days after the onset of symptoms. The swabs are then placed in a viral transport medium and can be kept for up to 72h at 2–8 °C, but should be stored below −70 °C for longer time [162] The rRT-PCR (real-time Reverse Transcription Polymerase Chain Reaction) assay, which relies on the recognition and amplification of viral RNA, is the “gold standard” for diagnosing COVID-19 [163,164]. The interpretation of rRT-PCR results is based on the number of amplifications that are necessary to obtain a detectable fluorescent signal, named cycle threshold (Ct). The Ct is inversely proportional to the viral load of the sample but does not correlate with the severity of the disease [164]. More recently, Rapid Antigen Tests (RATs) have emerged as low-cost, fast and simple-handling tests for COVID-19 diagnosis [165]. These tests detect viral antigens using specific recombinant antibodies. RATs are less sensitive than rRT-PCR assays because they can detect the presence of high loads of viral antigens, only when the patient is most infectious. Tests that are commercially available or in development for the diagnosis of COVID-19 are listed at the following address: https://www.finddx.org/covid-19/pipeline/ (accessed on 5 December 2020) [166].

Q24—What are the roles of the serological tests? [167,168]

While the diagnosis of SARS-CoV-2 infection (acute phase) is primarily based on detection of viral RNA (see Q23), serological tests, which detect SARS-CoV-2 specific antibodies (IgM, IgG and/or IgA), are used to identify exposure to the virus. Indeed, IgM and IgG are not detectable until 1–2 weeks following the onset of symptoms [72] (see Q14). Serological assays are mainly blood tests, but they can also be performed on other body fluids, including the oral fluid (see Q25). Different types of serological assays have been developed and include quantitative assays to determine antibodies titers (enzyme-linked immunosorbent assays (ELISAs)), assays with binary results (yes/no; lateral flow assays), and assays that show Ab functionality (virus neutralization assays). In ELISA and lateral flow assays, recombinant SARS-CoV-2 antigens (spike (S) and nucleocapsid (N) proteins, receptor-binding domain (RBD) domain of the S protein) are used to detect specific antibodies. Neutralization assays are more complicated to implement as they require the use of replication-competent infectious SARS-CoV-2 (biosafety level 3 facilities). The main purpose of serological tests is to measure the antibody responses induced by SARS-CoV-2, but also by the vaccination, and to determine seroconversion. Both quantitative and functional antibody assays will be important in evaluating immune protection against reinfection, and known protective titers would be extremely beneficial, in particular for vaccine development. Serological tests also play an essential role in epidemiological studies, to evaluate the prevalence of SARS-CoV-2 infection in different populations, and to determine the level of “herd immunity”.

Q25—What could be the benefits of using saliva tests?

Nasopharyngeal swab (NPS) has been recommended by the World Health Organization, especially to test early stage SARS-CoV-2 infection [169], but may be associated with pharynx irritation, pain, sneezing and cough, increasing the risk of contamination [170]. Saliva offers many advantages because its collection is easy, potentially carried out at-home by the patient, non-invasive, inexpensive, stress-free, painless, and with a minimal infection risk [171,172]. Saliva tests have also been developed and approved by the Food and Drug Administration (FDA) with an Emergency Use Authorization, as saliva contains SARS-CoV-2 (see Q30 and Q31). In fact, viral loads equivalent to those obtained from NPS are present in saliva the first week of symptoms, then decrease over time [173]. Based on the presence of viral RNA in saliva, but also of specific antibodies such as IgA (detectable 2 days after the onset of symptoms), some tests such as rRT-PCR or ELISA can be performed using saliva, but require a medical laboratory [172,174]. The promising role of saliva is highlighted by some tests that are usable in medical office as diagnostic tool, for example by colorimetric RT-LAMP (reverse transcription loop-mediated isothermal amplification) [175] or on the field (Point-Of-Need) for mass screening, in particular by lateral flow assay (Rapid Salivary Test), which detects the presence of the virus (Antigen Test), by identifying the spike (S) protein in saliva in a few minutes [176].

Q26—What are the diagnostic performances of saliva tests?

When comparing saliva with nasopharyngeal swab (NPS), the sensitivity values of salivary rRT-PCR ranged from 60% to 98% (mean sensitivity of 85%). Specificity values settled over 90% in most cases [172,177,178]. However, several studies have reported positive saliva samples from COVID-19 patients with negative NPS, suggesting that the combined use of saliva and NPS tests could increase diagnostic accuracy [172,178]. Detection of salivary IgA by ELISA tests, seems to show good diagnostic accuracy (>90% agreement with rRT-PCR) [174], as well as Point-of-Care technologies with RT-LAMP (95% agreement with rRT-PCR) [179] and Point-of-Need tools with Rapid Salivary Test (sensitivity of 93%) [176]. Further studies are needed, in particular for asymptomatic individuals, where the diagnostic accuracy of these tests is still largely under evaluation. However, these tests could be useful before aerosol-generating treatments and could reduce the risk of SARS-CoV-2 transmission in dental offices.

## 6. SARS-CoV-2 Transmission and Oral Cavity

Q27—Is the oral cavity a potential entry route for SARS-CoV-2?

The oral cavity can be a significant reservoir for respiratory pathogens such as *Mycobacterium tuberculosis*, Influenza virus, SARS-CoV, MERS-CoV, but also SARS-CoV-2 [180,181,182,183,184,185,186]. Several mechanisms could explain the ability of these oral pathogens to exacerbate lung infection including their oral inhalation into the lower respiratory tract, by swallowing contaminated oral fluid, but also by the oral localization of host receptor-proteases-mediated pathways facilitating their viral infectivity [184,187,188].

Q28—Which are the oral sites expressing receptor-proteases of SARS-CoV-2 infectivity? Other receptors?

**Figure 1:**
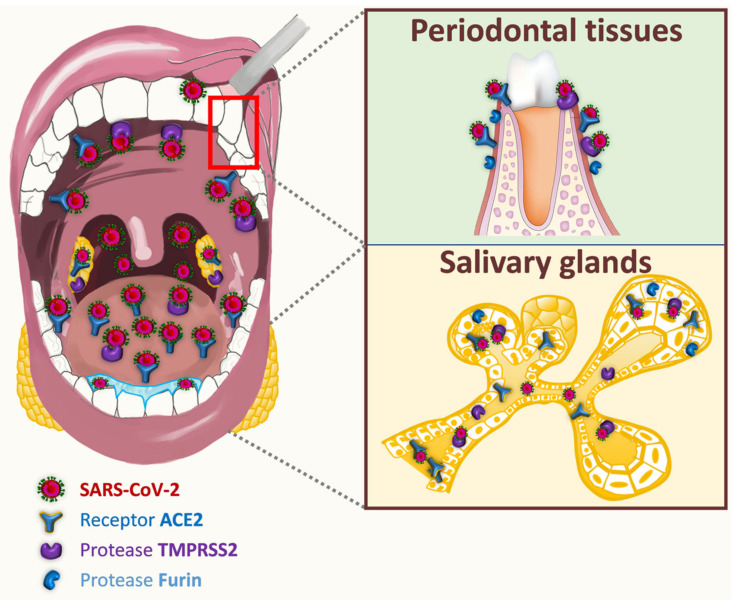
Potential entry routes for SARS-CoV-2.

The transmembrane protein receptor ACE2 (angiotensin 2 converting enzyme), as well as TMPRSS2 (transmembrane serine 2 protease) and furin enzymes, have been identified as critical determinants of oral SARS infectivity [189]. ACE2 is expressed on different cells of oral tissues including oral mucosa, gingiva, tongue, salivary glands, and tonsils [49,190,191,192,193,194,195,196] (Figure 1). Almost 96% of ACE2-positive oral cells would locate in dorsal tongue. Epithelial cells of the oral cavity showed abundant expression of ACE2 receptor, that is also expressed in T cells, B cells, and fibroblasts, although to a lesser extent [190,194]. ACE2 is reported to be predominantly localized to the basal cells of stratified squamous epithelium but was also visible in the horny layer of keratinized epithelium and finally, in tongue coating [49,190,191]. Interestingly, gingival sulcular epithelium tended to display stronger ACE2 expression than the buccal gingival epithelium [191]. The presence of ACE2 is confirmed in the taste epithelial cells of tongue fungiform papillae. The epithelial cells of salivary ducts and serous cells of human submandibular glands express abundantly ACE2 [191,195,196]. Its expression in epithelial cells of minor salivary glands is even higher than in lung cells, and could constitute a reservoir zone for SARS-CoV-2 in asymptomatic patients [193,196]. Interestingly, TMPRSS2 and furin were found to be expressed globally in the same oral tissues as ACE2 (dorsal tongue, gingiva, salivary glands, taste buds) [191,196,197]. TMPRSS2 is expressed in the squamous epithelium of the tonsils [198,199]. Oral localization of furin was not systematically associated with that of TMPRSS2 and ACE2. Furin-positive cells were neither observed on the surface of the squamous epithelium of the dorsal tongue and salivary ducts, nor on tongue coating. Conversely, furin was secreted in saliva like TMPRSS2 [191]. TMPRSS2 may play a larger role in oral infection compared to furin, and ACE2–TMPRSS2 co-expression is a privileged target for SARS-CoV-2 infection. 

The membrane protein neuropilin (NRP1) and extracellular MMP inducer (EMMPRIN) have been recently considered as other targets for SARS-CoV-2 infectivity. NRP1 is expressed in the differentiated epithelial cell layers of human normal tongue and in epithelial cells of human healthy salivary glands. The neuropilin-1 receptor is up-regulated in dysplastic epithelium and oral squamous cell carcinoma [200,201,202,203,204]. EMMPRIN expression is also up regulated in oral squamous cell carcinoma. Since ACE2 expression is depleted in oral squamous cell carcinoma, EMMPRIN receptor might be taken over for SARS-CoV-2 entry into cancer host cells [201,205]. The oral expression of all these factors indicate that oral cavity may be vulnerable to SARS-CoV-2 invasion.

Q29—Does SARS-CoV-2 penetrate the oral tissues?

While Wang et al. have reported a proliferation of SARS-CoV in exfoliated epithelial cells in saliva [184], SARS-CoV-2 is detected with a sensitivity of 89.8% on the surface of the tongue after swabbing [206]. To our knowledge, there is only one article demonstrating the direct presence of SARS-CoV-2 in COVID-19 autopsy oral tissues such as human salivary glands and mucosa. In particular, SARS-CoV-2 was detected in oral squamous keratinocytes [196]. 

Dysgeusia and xerostomia (early symptoms associated with SARS-CoV-2 infection) [195,207,208,209], but also some oral manifestations such as tongue ulcers [210], could be related to the presence of SARS-CoV-2 invasion factors (such as ACE2 and TMPRSS2) on the taste buds and dorsal tongue [196]. Interestingly, the expression of ACE2 and TMPPRSS2 in gingival sulcular epithelium (directly linked to gingivitis or periodontitis) [191], and the detection of SARS-CoV-2 in the inflammatory gingival crevicular fluid [211], raise questions on the possible role of this epithelium in SARS-CoV-2 infection. The potential passage of SARS-CoV-2 through the systemic route [212] could be considered as it has been demonstrated for periodontal bacteria such as *Porphyromonas gingivalis* [213]. It might be possible to imagine the risk of co-infection between SARS-CoV-2 and bacteria of the periodontal pocket. Co-infection of influenza virus and *Porphyromonas gingivalis* could initiate in vitro the autophagy of pulmonary epithelial cells [214].

Q30—How does saliva represent a reservoir for SARS-CoV-2?

Whole saliva is a biological fluid secreted by major and minor salivary glands and contains gingival crevicular fluid (GCF), desquamated oral epithelial cells, dental plaque, bacteria, nasal and bronchial secretions, blood and exogenous substances [215]. The detection of SARS-CoV-2 in saliva was first reported in 11 COVID-19 patients (91.7%) in Hong Kong [216]. Since then, more than 250 publications have revealed the presence of SARS-CoV-2 in saliva, in connection with the development of saliva diagnostic tests for COVID-19. At least four different pathways for SARS-CoV-2 entry are suggested into saliva: first, by major and minor salivary gland infection; second, from the lower and upper respiratory tract (sputum, oropharynx, cough); third, from the blood into the GCF and fourth, from dorsal tongue [206,217]. Since SARS-CoV has been shown to be able to infect epithelial cells in salivary gland ducts, as early as 48h after its intranasal inoculation in rhesus macaques [192], autopsy of human salivary glands from COVID-19 patients confirmed SARS-CoV-2 infection in these tissues [196]. Furthermore, SARS-CoV-2 nucleic acids were detected in pure saliva from mandibular salivary glands [195]. The salivary glands could constitute a direct source of the virions in the saliva. Saliva is principally secreted from the salivary glands but can contain secretions coming down from the nasopharynx or from the lung, especially later in infection. Saliva samples obtained by coughing up saliva from the posterior oropharynx, were collected from 23 SARS-CoV-2 infected patients. Of these, 87% were tested positive for SARS-CoV-2 [216]. Yet, it is possible that these samples included secretions from the nasopharynx or lower respiratory tract. A passive contamination of sputum could affect the kinetics of saliva [218,219]. Some SARS-CoV-2 positive ciliated cells originating from nasal cavity are found in the saliva [196]. SARS-CoV-2 infected GCF establishes the possible contribution of this fluid to the viral load of saliva [211]. Finally, the presence of SARS-CoV-2 on the dorsal tongue and in infected squamous epithelial cells in saliva [196,206] provides a potential cellular mechanism for spread and transmission of SARS-CoV-2 by saliva.

Q31—How does the profile of the viral load in oral fluid change over time?

SARS-CoV-2 viral RNA load in oral fluid globally ranged from 9.9 × 10^2^ to 7.1 × 10^10^ copies/mL [161,173,176,216,220,221,222,223,224]. The peak was globally reached during the first week of symptom onset and declined over time with gradual symptom improvement [161,173,183,216,220,221,222,223,225,226]. A high load in the pre-symptomatic phase could also be expected [227]. During the period of virus shedding, viral RNA could be detected up to 25 days after symptom onset [161,173,184,216,219] and in one case report, up to 37 days [228], independently of the severity of the illness [184]. Few studies have reported an association between viral loads and severe symptoms [173,216,225,229]. Although in a study using posterior oropharyngeal saliva, viral loads were found higher (1 log10 higher) in patients with severe disease compared to patients with mild disease, this relationship was not statistically significant [216]. No significant difference was observed in disease severity or clinical symptoms between patients in whose saliva viral RNA was detected or undetected [225]. However, the prevalence of severe disease and cough were frequently higher in patients in whom viral RNA from saliva was detected [218]. Interestingly, several studies have reported the presence of viral RNA in the saliva of asymptomatic patients [220,225,230,231,232]. Salivary SARS-CoV-2 RNA was detected in more than 50% of asymptomatic patients and of patients before the symptom onset [225]. Among 98 asymptomatic health-care workers, two individuals were tested negative for matching self-collected nasopharyngeal samples, but positive in saliva [161]. Alternatively, saliva samples from symptomatic patients with negative SARS-CoV-2 NPS could also be positive [233,234]. Saliva may be more sensitive in detecting asymptomatic or pre-symptomatic infections. The timing and duration of infectivity are important to establish, especially for asymptomatic individuals, because the risk of transmission by air through salivary droplets is possible. Indeed, the relationship between SARS-CoV-2 detection, viral load and infectivity is still unclear as viral RNA may not represent infectious transmissible virus. Viral culture studies using COVID-19 patients to confirm the presence of infectious SARS-CoV-2 are limited. A positive viral culture of infectious virus was found from the saliva of three patients [221]. The infectivity of SARS-CoV-2 in saliva has been demonstrated, even 15 days after the onset of clinical symptoms, using cell culture and an animal model [235]. A recent study suggested that no viable virus could be cultured from salivary swab specimens collected from COVID-19 patients with prolonged viral RNA shedding (>20 days after diagnosis) [236]. The risk of virus transmission can therefore be expected to be low, even though late viral shedding is present in asymptomatic or mildly symptomatic patients. Further investigations with larger cohorts and standardized procedures are necessary to precise the correlation between salivary viral loads, disease severity, infectivity of salivary virus.

Q32—What are the physiological aerosolization mechanisms of oral and nasal fluids?

**Figure 2:**
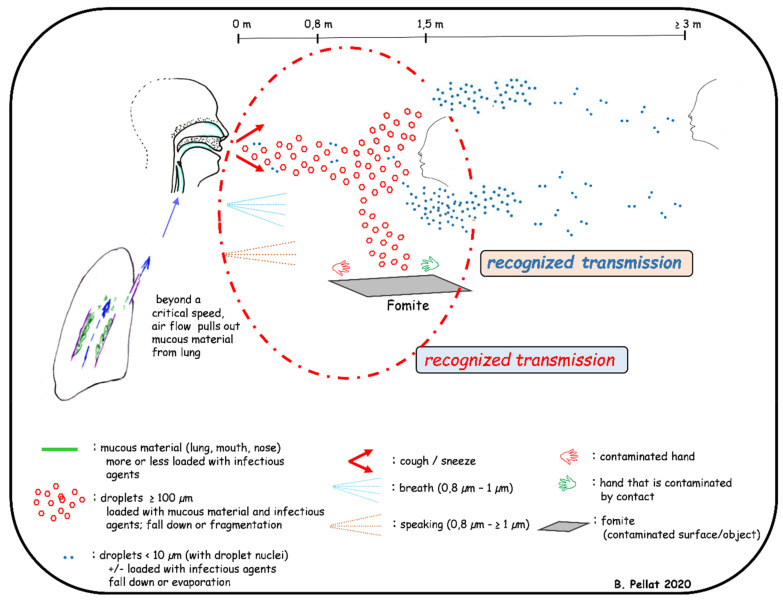
Aerosolization mechanisms of oral and nasal fluids.

SARS-CoV-2 is transmitted to human either by hand carriage or by airborne route. In both cases, the virus originates from nose and/or mouth of an infected patient when breathing, speaking, sneezing, coughing or during dental treatments. By breathing, the warm (36 °C) and moist (6.2% water) gases produced in alveoli rise to the mouth and nose where they cool and condense before being expelled (0.6 to 1.4 m/s) in the form of droplets by the respiratory flow. These droplets (0.8–1 µm diameter) contain water and mucous particles from the alveolar and the upper respiratory tract, and the eventual infectious agents. They form a bio-aerosol and can contaminate nearby people but can also remain in the atmosphere (Figure 2). The questions of virus viability duration and concentration in air remain unsolved [237]. Speaking differs by the vibrations of the vocal cords, the longer exhalation time, and the typical flow and pression due to some consonants. Thus, droplets are sprayed from 0.5 to 3 m with possible contamination (Figure 2). The same question of virus viability duration and concentration in the air remains [238]. By coughing and sneezing, air expulsion is brutal (up to 13 m/s), resulting in the transport of a large amount of alveolar, nasal/oral mucous materials and infectious agents included in very large droplets up to 100 µm [239]. In a few milliseconds, the droplets flatten and split up over a distance of 0.7 m. The heaviest particles fall down and contaminate the underlying surfaces which become fomites. In 10–20 s, the largest droplets lose water through evaporation, mostly in case of low relative humidity and high atmospheric temperature [240]. The resulting little particles with a low water content (i.e., droplet nuclei) and can stay in the atmosphere for many hours or even days (Figure 2). The aerial viral load can therefore increase over time, mostly in closed spaces without sufficient ventilation. Inhaled airborne viruses deposit directly into the human respiration tract. Finally, airborne transmission appears to be highly virulent and represents an important transmission route of the disease [241]. 

Q33—How is indirect viral transmission by fomites possible for COVID-19?

Direct droplet and airborne transmissions of SARS-CoV-2 occur at variable distance and extended duration [242]. The droplets and droplet nuclei containing SARS-CoV-2 fall down (<1.5 m and several meters, respectively) and contaminate the surrounding surfaces which become fomites (Figure 2). Viral transmission from contaminated surfaces or fomites has a long history, including self-inoculation of the oral, nasal and ocular mucous membranes by hands that have touched these surfaces [243]. This transmission route is important in dental settings where aerosolization of droplets containing SARS-CoV-2 is also generated by many dental instruments. The bio-aerosols produced could be found several meters from the patient’s mouth and could remain in the atmosphere of the treatment room for several hours before settling on the worktops [237].

Q34—How long can an infected surface remain contaminated?

Regarding the stability of viruses on surfaces, the persistence of SARS-CoV-2 infectivity on fomites has been analyzed by spraying a solution containing the virus onto various surfaces [244]. The stability is higher on plastic and stainless-steel surfaces, 72 h and 48 h, respectively, than on copper and cardboard, 4 h and 24 h respectively. Another study showed that internal and external protective masks may be contaminated for several days with SARS-CoV-2 [245]. These results increase the probability of transmission by contact with fomites since the virus can remain viable several days on supports (plastic, steel) that frequently found in the medical environment [245,246].

## 7. Clinical Presentation of COVID-19 and Risk Factors 

Q35—What are the main presenting symptoms of COVID-19?

Many individual variabilities in the clinical manifestations of COVID-19 have been recorded, ranging from asymptomatic patients confirmed by rRT-PCR to severe forms of infection. The mean incubation period has been reported to be around 5.44 days [247]. The differences in clinical features are due to the age of the infected individuals, their underlying conditions, immune status, coinfection, or even the daily diet, which seems to alter ACE2 expression [248]. 

World Health Organization (WHO) has classified three levels of symptoms [23].

- Most common symptoms: fever, dry cough and tiredness.
- Less frequent symptoms: loss of taste or smell, nasal congestion, conjunctivitis, sore throat, headache, muscle or joint pain, skin rash, nausea/vomiting, diarrhea, chills or dizziness.
- Severe manifestations: shortness of breath, loss of appetite, confusion, persistent chest pain or pressure, high temperature (above 38 °C) that can lead to acute respiratory distress syndrome and “cytokine storm” (see Q18).

Some symptoms may persist, collectively referred as post-COVID syndrome, such as tiredness, cough, congestion, shortness of breath or even loss of taste or smell [249,250]. Additionally, COVID-19 may increase the risk of health problems by affecting certain organs such as the heart or lungs.

In the oral cavity, sudden loss of taste and smell has been suggested as an early and easy indicator of COVID-19 [251,252]. 

Q36—What are the main comorbidities and risk factors of COVID-19?

The links between COVID-19 severity and the presence of underlying comorbidities have been thoroughly studied [71,253,254]. Richardson et al. have concluded that hypertension, obesity (see Q38), diabetes and chronic obstructive pulmonary diseases are the most common comorbidities [255]. The risk is increased in elderly patients with weakened immune response, higher frequency of metabolic syndrome along with an increased damage of endothelial cells, as well as increased affinity and distribution of ACE2 (angiotensin 2 converting enzyme) and TMPRSS2 (transmembrane serine 2 protease) compared to children [256,257]. Stable vitamin D3 level and melatonin availability may have protective effects against COVID-19 [258,259]. Smoking and exposure to nicotine, associated with the fragility of the cardiopulmonary system, may be linked to severe COVID-19 forms. However, some studies have suggested a protective effects of smoking via the anti-inflammatory action of nicotine [260,261]. Drug–drug interactions (especially in the context of cancer and autoimmune diseases) have been also considered as a major factor affecting the circuit of COVID-19 for patients receiving these therapies [262]. The severe forms of COVID-19 in patients with underlying conditions have been explained by the availability of ACE2 in different organs (including lungs, heart, kidneys, brain and oral mucosa), the extreme immune reaction to SARS-CoV-2 (see Q18), and also the variations of microbiota (see Q22) [253,263,264,265]. In the oral cavity, oral submucous fibrosis seems to worsen COVID-19 by activating ACE2 [266]. Poor oral health, as an indirect cause of comorbidities, may increase the risk of severe symptoms [267].

Q37—What are the main symptoms in children and adolescents? Can they present severe forms of COVID-19?

COVID-19 is much less common in the pediatric population. In a cohort of 44,672 confirmed cases, only 2% were children and adolescents aged from 0 to 19 years [87]. Severe forms are rare within this population (0.6%) [268] with very low morbidity and mortality rates compared to the adult population (0.3% of total deaths in the US) [23]. Children tend to develop a milder disease with reduced respiratory symptoms and a very low incidence of acute respiratory distress syndrome (ARDS). Although a large proportion of infected children is asymptomatic [87,90,91], they can spread SARS-CoV-2 [269]. COVID-19 can affect children at all ages (average age: 8–9 years) with no significant sex difference [270]. Children have been typically exposed to the virus through a family member (75.6%) [271]. Fever remains the main presenting symptom together with cough, rhinorrhea and tiredness [271]. 

Children with other underlying conditions (e.g., congenital heart diseases, pulmonary chronic diseases, diabetes, immune-related disorders, co-infections, obesity) may however develop severe forms of COVID-19 [136,270]. In rare cases, SARS-CoV2 infection has also been associated with severe multisystem inflammatory syndrome (MIS-C or Kawasaki-like hyperinflammatory syndrome) in previously healthy children [272]. 

No evidence of any related oral manifestation of SARS-CoV-2 infection has been found. All reported manifestations, like fissured lips, erythema, or strawberry tongue (Kawasaki-like disease manifestations) were more related to the underlying conditions and the immune system rather than to the infection itself [273]. 

Maternal–fetal transmission of COVID-19 during pregnancy is about 2.67% [274], but it is unknown whether the newborns were infected during pregnancy or delivery [275]. SARS-CoV-2 infection during pregnancy seems to be associated with a higher disease severity and an increased frequency of fetal and neonatal complications [276]. However, no relationship between the exposure of newborns to SARS-CoV-2 and the severity of COVID-19 is yet well established [274].

Q38—What are the links between COVID-19, overweight and malnutrition?

The links between COVID-19, weight and nutrition are complex. On the one hand, in Europe, the first lockdown resulted in weight gain in approximately 30–40% of the population (average 2.5–3.0 kg) [277,278]. This was due to boredom or stress, resulting in an increase in calorie intake (overeating, alcohol) associated with limited outdoor exercise [279]. Besides, overweight people have an increased risk to develop a severe or lethal form of COVID-19 (see Q36) [280]. On the other hand, lockdown resulted in weight loss in approximately 10–20% of the population, in average 3 kg [278]. Loss of appetite was due to stress (fear of going out, income decrease), social isolation or a depressed state [279]. In addition, approximately 60% of people with mild to moderate forms of COVID-19 have anosmia and ageusia, which generally regress within a few weeks [281,282]. Severe or persistent forms can cause anorexia and rapid weight loss. Whether it is recent or installed, underweight is usually the sign of protein-energy malnutrition. SARS-CoV-2 infection is characterized by inflammatory syndrome leading to increased muscle catabolism and increased protein-energy needs. There is a vicious circle, because dyspnea, oxygen therapy and isolation hinder food intake [283]. In a study involving 403 patients hospitalized for COVID-19, 70% of them left the hospital with malnutrition and an average loss of 6.5 kg [284].

Q39—What are the main oral manifestations of COVID-19?

Taste impairment is considered to be one of the most common oral manifestations directly linked to SARS-CoV-2 infection, with different degrees varying from dysgeusia, hypogeusia, to ageusia [285,286]. Taste alterations can be one of the earliest signs of COVID-19 and may be the only symptom of COVID-19 in asymptomatic and mild forms of the disease [287]. Prevalence variations of taste disorders have been reported between populations [288] but no significant sex difference has been found [289]. Taste disorders seemed to affect older and hospitalized patients [290], but they can affect younger patients too [289]. First, it was proposed that taste disorders may be associated with olfactory dysfunction [289], but later with increasing case reports, it has been shown that they may happen with or without, and even before the apparition of olfactory disorders [291]. No significant association has been found between comorbidities and the development of olfactory or gustatory dysfunctions [289]. Dysgeusia was also linked to poor oral hygiene and hyposalivation [290]. Many difficulties in evaluating this dysfunction have been reported and include the lack of specific tests, the fact that some COVID-19 patients did not remember having taste disorders and that patients with severe forms were not evaluated for dysgeusia. The four taste receptors (i.e., salty, sweet, bitter, sour) can be affected [289]. Many hypotheses have been proposed to explain taste disorders in COVID-19 patients [291,292,293]. They may result from interactions between neurons expressing high levels of ACE2 and SARS-CoV-2, which consequently disturb the gustatory pathway by affecting gustatory cranial nerves (VII, IX, X) [292]. The tongue and taste buds’ cells that highly express ACE2, interact with SARS-CoV-2, and facilitate its tissular invasion, subsequently altering taste function (see Q28). This was explained by the dysregulation of dopamine and serotonin pathway [292]. This hypothesis is based on previous findings on taste impairment with ACE inhibitors used to treat hypertension [294]. Taste disorders have also been considered as a side effect of COVID-19 treatment [292]. Finally, it has been suggested that SARS-CoV-2 binds to sialic acids of salivary mucins, which leads to their accelerated degradation and the alteration of gustative function [195]. Despite the absence of evidence, dysgeusia seems to persist in some patients, even after COVID-19 recovery [207]. 

Alteration of salivary glands secretion have also been reported in COVID-19 patients (about 30% of hospitalized patients) [295] but the links are not yet well established. Elderly patients and patients with other comorbidities such as hypertension or diabetes have pre-existing decreased salivary secretion, which makes it difficult to perform an objective reliable evaluation. Since ACE2 is expressed by acinar epithelial cells of major and minor salivary glands [221], some authors have hypothesized the development of acute sialadenitis during SARS-CoV-2 infection phase and chronic sialadenitis after recovery [296]. This hypothesis has been supported by series of case reports of acute parotitis and submandibular gland sialadenitis in middle-aged to elderly COVID-19 patients [297,298,299]. Altogether, this supports a possible direct link between SARS-CoV-2 infection and sialadenitis, but further investigations are needed in order to establish this relationship, such as eliminating all other viral co-infections of salivary glands and expand clinical observations to larger cohorts of COVID-19 patients. 

Some authors have described oral manifestations close to those associated with other oral viral infections such as oral pain (burning), desquamative gingivitis, irregular ulcers and blisters, aphthous stomatitis, glossitis, mucositis, patchy tongue, recurrent herpetic stomatitis, lip semi mucosa or vesiculobullous lesions [300,301,302,303]. Increased stress and tiredness during COVID-19 course have been associated with an increased risk of developing other oral viruses like Herpes simplex virus or Varicella-zoster virus [304]. ACE2, TMPRSS2 (transmembrane serine 2 protease) and FURIN proteins are highly expressed by epithelial cells of different oral mucosae (see Q28) [190]. Despite the low number of reported cases of these manifestations, it seems that they equally affect men and women. All oral mucosa localizations were found (tongue, palate, lips, gingiva, buccal mucosa). In mild cases, oral mucosal lesions developed before or at the same time as the initial respiratory symptoms. Viral exanthem was also suggested to be a COVID-19 related clinical manifestation [305]. Due to lockdown and altered lifestyle (poor oral health or overconsumption of mouthwashes, tobacco, alcohol), some oral mucosa pathologies could find suitable conditions for their development or recurrence. Some oral manifestations such as candidiasis have been reported to be due to opportunistic infections caused by broad spectrum antibiotics prescription [306]. Similarly, halitosis was described and associated to epithelial changes of keratinized tongue desquamation [307]. Variation of oral clinical manifestations may be found even between different members of the same family infected with SARS-CoV-2 [308]. Altogether, this suggests that oral mucosal lesions should be thoroughly investigated in COVID-19 patients.

Q40—What is the impact of COVID-19 on patients with rare diseases?

As the majority of rare diseases are chronic, COVID-19 pandemic has exacerbated the difficulties encountered by this population, from potential reduced access to medical care to increased anxiety, with a significant impact on their health status and social well-being [309]. Due to the very wide number of rare diseases (over 7000) and their great variability, it is not possible to address here the impact of COVID-19 on each of these rare conditions. Expert recommendations and information regarding COVID-19 and specific rare diseases are available at the following address: http://international.orphanews.org/summary/id-200327.html (accessed on 21 January 2021).

## 8. Therapeutic Management of Patients with COVID-19 

Q41—Which treatments have been proposed for COVID-19?

Early in the course of the infection, the disease is driven by SARS-CoV-2 replication. At advanced stages, the disease is driven by an excessive inflammatory response to the virus, leading to immune-mediated tissue damage, particularly in the context of concomitant “cytokine storm” (see Q18). It has been hypothesized that antiviral strategies would be more effective in the early course of disease, while immunosuppressive therapies may be beneficial in the later stages of COVID-19.

The National Institute of Health provides treatment guidelines available at: https://www.covid19treatmentguidelines.nih.gov/ (accessed on 6 December 2020) [310].

**Figure 3:**
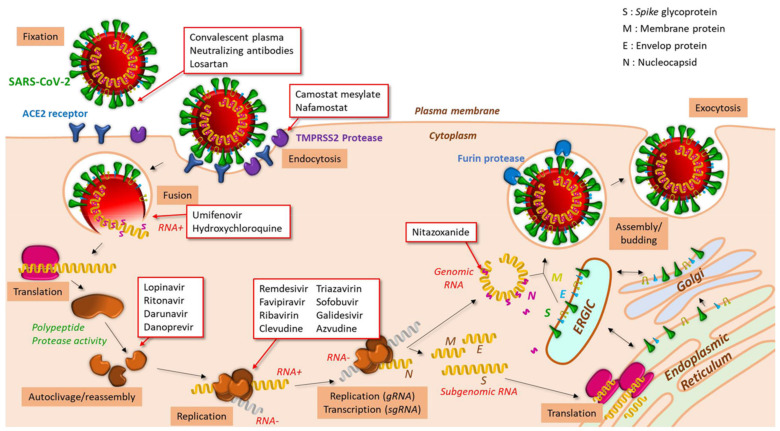
Mechanisms of action of the antiviral drugs on the viral life cycle.

Several antiviral strategies have been proposed and almost all steps of viral replication have been targeted. All registered clinical trials using antiviral strategies against SARS-CoV-2 have been reviewed [311]. Chemical molecules tested in clinical trials are gathered in Table 1 and the mechanisms of action of these antivirals on viral life cycle are shown in Figure 3.

- Serotherapies, based on the transfusion of plasma coming from convalescent patients have early been proposed [312]. This strategy assumes that convalescent plasma contains a cocktail of neutralizing antibodies against SARS-CoV-2.
- Bamlanivimab is a monoclonal antibody-based therapy, using neutralizing IgG1 targeting the receptor-binding domain (RBD) of the spike (S) protein from SARS-CoV-2. Clinical trial showed a reduction of hospitalizations for COVID-19 during the 28 days after treatment, with an improvement of symptoms [313].
- Chemical drugs (Table 1) target the different steps of the virus life cycle, from entry to virion assembly. Most of the drugs that have been tested in trials are antiviral molecules that had been developed against other viruses and reused in the fight against SARS-CoV-2.
- Type I interferons (IFN) are antiviral cytokines that have shown efficacy in the treatment of several viral diseases They trigger the regulation of more than 1000 genes involved in adaptive or innate immunity, allowing the infected cell to enter in an antiviral state, decreasing viral spreading, upregulating antigen presentation and recognition by T and B cells. While type I IFN pathways are targeted and inhibited by SARS-CoV-2 (see Q13) [314], the virus appears to be sensitive to treatment with exogenous IFN-β and IFN-α2. Hence, several clinical trials were conducted using type I IFN alone, or in association with other drugs, showing a decrease of severe symptoms or a lower mortality [315].

Several immunosuppressive therapies are currently under investigation or at various phases of development to control or prevent the development of “cytokine storm” syndrome [59,137] (see Q18). Treatment with dexamethasone, a corticosteroid, has been shown to improve survival in patients with severe COVID-19 and receiving respiratory support [334]. Therefore, the use of dexamethasone has been strongly recommended [334,335].

COVID-19 has been associated with a prothrombotic state [336] and an increased incidence of thromboembolic disease has been reported [337]. Anticoagulant thromboprophylaxis has been recommended (in the absence of a contraindication) in acutely/critically ill hospitalized patients by different expert panels [338,339,340]. However, the risks and benefits of anticoagulation in COVID-19 patients must be evaluated by dedicated clinical trials.

## 9. Vaccine Strategies for COVID-19

At the beginning of January 2021, more than 60 candidate vaccines reached the clinical trial stage of development. Out of them, 10 reached phase III, and 5 were used for vaccination in various countries. World Health Organization maintains a landscape document referencing the candidate vaccines in development [341], available at: https://www.who.int/publications/m/item/draft-landscape-of-covid-19-candidate-vaccines (accessed on 22 November 2020).

Q42—Which are the various strategies to design vaccines to protect against SARS-CoV-2 infection? [81,342,343,344]

Several vaccine platforms are under development and include:

- Inactivated virus vaccines: They are produced by culturing SARS-CoV-2 in cell cultures followed by inactivation of the viral particles to prevent their replication into the host. Whole virus or subunits may be used. Three candidates are in phase III, and 5 candidates are in phases I/II.
- Viral vectored vaccines: They use viral vectors (i.e., another virus than SARS-CoV-2) engineered to express SARS-CoV-2 proteins and able to infect target cells. The latter produce viral proteins that usually induce strong humoral and cellular immunity. Non-replicating human or simian adenoviruses are used as viral vectors in several clinical trials (four in phase III). Replicating viral vectors from vesicular stomatitis virus or measles virus are also used for the development of COVID-19 vaccines (currently in phases I/II).
- Protein and peptide vaccines: Recombinant SARS-CoV-2 proteins or peptides may be used for vaccine formulations. Candidate vaccines focus on the S protein or its RBD domain subunit to obtain antibodies that neutralize virus entry in target cells. Fifteen candidates are in phases I/II, and 4 in phases II/III.
- mRNA vaccines: Viral protein-specific mRNA encapsulated into lipid nanoparticle are expected to reach the cytoplasm of target cells. Thus, cells produce and release the protein of interest, which induces both humoral and cellular immune responses. This technology is new, and mRNA vaccines pose logistical issues as they need to be stored at very low temperatures (−80 °C). Two mRNA vaccines encoding the S glycoprotein or its RBD subunit were claimed to be at least 90% protective against COVID-19 as a result of the phase III trials. Four other mRNA vaccines are under phase I/II clinical trials.
- DNA vaccines: They are based on a plasmid DNA containing the gene of the S protein or its subunits under the control of a mammalian promoter. Despite the high stability of plasmid DNA, DNA vaccines often exhibit low immunogenicity, and have to be administered via delivery devices (e.g., electroporators) to make them efficient. Yet, no DNA vaccine reached the phase III, but five are in phase I/II.

Q43—How to control vaccinal efficiency and safety? [81,343,345]

The efficiency and safety of a candidate vaccine are supported by several properties: (1) a virus-specific immunogenic preparation inducing long-term protection, (2) limited and controlled side-effects, (3) storage conditions that allow an easy distribution all around the world, (4) an easy route of administration that prevents infectious risks. Each antigenic formulation (see Q42) has interests and limitations for combining immunogenicity and tolerance. Immunogenicity is closely related to vaccine design and the presence of adjuvant. However, the adjuvant may vary depending on the route of administration (i.e., intramuscular versus mucosal). After the assessment of efficacy by in vitro and animal experiments, the efficacy and safety of a candidate vaccine for humans is determined by the 3-phase clinical trials. Phase I evaluates the safety of vaccine candidates on a limited cohort, phase II establishes formulation and dosages to optimize efficacy and to limit side-effects, and phase III demonstrates efficacy and safety in a larger cohort. In traditional vaccines development these clinical trials take 5 to 7 years, whereas they only took several months in the accelerated anti SARS-CoV-2 vaccines development. We have to keep in mind that the efficacy of a vaccine may be evaluated not only by total prevention of the disease but also by preventing the severe forms and decreasing the hospitalization rate. All vaccines that have reached phase III use the intramuscular route of delivery, which can limit their use in developing countries. However, candidate vaccines using mucosal routes are under investigation (see Q45).

Q44—What does “Vaccine-Associated Disease Enhancement” mean? [81,343,346,347]

Vaccine-associated disease enhancement (VADE) can result from Antibody-associated Disease Enhancement (ADE) and/or a Th_2_ biased immune response. ADE appears when the immune response produces low titers of neutralizing IgG antibodies. Thus, the antibody response is unable to block virus entry into target cells but can even facilitate it. Antigen-Ab complexes induce the release of inflammatory cytokines by binding to Fcγ receptors on immune cells, or by activating the complement cascade. In a similar way, the bias of the helper T-cell response to Th_2_ rather than to the anti-viral protective Th_1_ dominant response (cell-mediated response), induces pro-inflammatory cytokines release and eosinophilic infiltration. VADE results in an increased disease severity in vaccinated animals/humans submitted to natural infection. VADE has been reported during the development of several vaccines (against Respiratory Syncytial Virus, Dengue, SARS-CoV, MERS-CoV). To control the risk of VADE, SARS-CoV-2 candidate vaccines must induce (1) high and long-lasting titers of neutralizing antibodies, (2) low titers of non-neutralizing antibodies, and (3) a strong cellular immunity. More than likely, candidate vaccines entering phase III respond to these criteria. However, the diversity of the immune responses among the population (i.e., younger versus older, male versus female, previously infected versus naïve) and its impact as regards the risk of developing VADE is still an open question. Even if phase III trials have not evidenced such side effects, the exposure of vaccinated individuals to natural infection is not easy to follow, and probably, waiting for longer periods as well as larger cohorts will be needed to evaluate the real risk.

Q45—How could oral mucosal immunity contribute to vaccine development? [155,348]

Mucosal (nasal or oral) route vaccines for COVID-19 prevention represent 5 out of the 51 vaccines in clinical trials (December 2020). The nasal/oral routes present several interests for vaccine development against viral diseases, especially those affecting the airways: (1) secretory IgAs are polymeric and efficiently neutralize virus entry in animal models of SARS, (2) nasal/oral vaccines are associated with high titers of secretory IgA and a local cytotoxic T lymphocytes activation that may prevent severe forms of respiratory diseases, (3) unlike IgG, IgA are not able to activate Fcγ receptors expressing cells or the complement cascade and thus may limit the risk of a “cytokine storm” or ADE (see Q18 and Q44), (4) mucosal vaccines are easy to administrate, do not need medical training and prevent the risks associated with needle use. However, the mucosal immune system is devoted to maintaining homeostasis through non-inflammatory processes called “immune exclusion”. This immune exclusion tolerates the healthy microbiome and prevents tissue infection by pathogens. The stimulation of the mucosal immune system may induce tolerance rather than an active immunization, and the development of mucosal vaccines needs specific adjuvants.

## 10. Infection Prevention and Control in Dental Facilities Based on World Health Organization (WHO), European Centre for Disease Prevention and Control (ECDC) and Centers for Disease Control and Prevention (CDC) Recommendations

### 10.1. Identification and Management of Suspected/Confirmed COVID-19 Patients

Q46—How to identify suspected/confirmed patients with COVID-19?

Suspected COVID-19 patients are symptomatic patients showing signs of COVID-19 (see Q35) or asymptomatic patients in close contact—within the previous 14 days—with another person infected or presenting these symptoms [349]. Confirmed COVID-19 patients are symptomatic or asymptomatic patients who have been tested positive for SARS-CoV-2 with rRT-PCR or rapid antigen test [169]. The early and rapid recognition of infected patients and patients in close contact with COVID-19 infected individuals aims at limiting contacts with others to break the viral chains of transmission [349]. Screening questionnaire based on the criteria of confirmed/suspected SARS-CoV-2 infection should be carried out by telephone or by internet when a patient makes an appointment, and at the dental office entrance [350,351]. 

Q47—How to manage dental appointments?

Patients should access the dental office only by appointment [350]. To minimize contact with other patients, only one single patient is ideally allowed in the waiting room with waiting time as short as possible [350,352]. The planning schedule should be set with sufficient time for patients’ appointments [350,351,353]. During the COVID-19 outbreak, patients should not be accompanied to the dental office unless necessary. Only essential persons such as parents of pediatric patients and guardian of patients presenting intellectual disability are allowed [350,351,352]. The presence of these persons is prohibited (if possible) during aerosol-generating procedures (AGPs) [352]. Patients should have their appointment be rescheduled if they show symptoms of COVID-19 within 10 days, if they have been tested positive for SARS-CoV-2 infection within 10 days, or if they have had close contact with a suspected/confirmed COVID-19 person within 14 days, prior to their scheduled appointment [353]. In case of dental emergency, their appointment must be set at the end of the day [351].

Q48—How to manage patients according to their COVID-19 status?

For patients who seem to be “negative” for COVID-19, all dental cares can be provided by applying the standard precautions and using a respirator for aerosol-generating procedures (AGPs). Patients with suspected/confirmed COVID-19 should not enter the dental facility, unless they need urgent dental care [350]. Only dental emergency should be handled minimally invasively—without AGPs if possible—in a well-ventilated room. The dental staff in the treatment room should be limited to essential personnel and the doors should always remain closed during treatment. Dental staff should apply standard, contact and droplet precautions when performing clinical exam, and add airborne precautions when performing AGPs (see Section 10.3 and Section 10.4) [349,351,352]. Tele-dentistry (i.e., telephone consultations or videoconferencing) could be an alternative to face-to-face outpatient visits, providing clinical support and pharmacological treatments without direct contact with suspected/confirmed COVID-19 patients [352]. An appointment can be made after the contagiousness period (see Q47 and Q57).

### 10.2. Identification and Management of Suspected/Confirmed COVID-19 Dental Staff Members

Q49—How to identify a dental staff member infected with SARS-CoV-2?

Early detection of SARS-CoV-2 infection among dental staff members may be achieved through daily self-assessment for signs and symptoms of COVID-19 [351,354], and laboratory testing in case of suspected SARS-CoV-2 contamination [354].

Q50—What to do if a dental staff member is suspected/confirmed COVID-19?

Dental staff members exposed to SARS-CoV-2—due to a close contact with a COVID-19 person without appropriate personal protective equipment—should be excluded from work, self-monitor their symptoms and self-quarantine for 14 days [353,355,356]. They should be tested [353,355]. A rRT-PCR test on day 10 after exposure can be performed and if it is negative, quarantine can be discontinued earlier [356]. Dental staff member presenting symptoms that are compatible with COVID-19 should stop working, self-isolate at home [350,351,353] and get tested [350,355]. A dental staff member with a positive SARS-CoV-2 test—with or without symptoms—should self-isolate at home. The safe return to work can be achieved after at least 10 days (minimum 20 days for severe COVID-19 and for immunocompromised staff member) with an additional 24 to 72h without fever associated with improvement of respiratory symptoms [354,357].

### 10.3. Applying Standard Precautions for All Patients in a COVID-19 Context

Q51—What are standard precautions?

Standard precautions are designed to reduce the risk of pathogen transmission, including bloodborne and airborne pathogens. They include hand and respiratory hygiene, use of appropriate personal protective equipment based on the risk assessment [351] (see Section 10.5), care equipment and environmental cleaning, and safe waste management [358].

Q52—How to perform hand hygiene?

Hand hygiene is one of the most effective method to prevent pathogen transmission and healthcare-associated infections [358,359], including COVID-19 [353]. Dental staff members should apply WHO’s “My five moments for hand hygiene” approach: before touching a patient, before a clean or aseptic procedure, after body fluid exposure risk, after touching a patient, and after touching patient surroundings (whether or not gloves are worn). In addition, hand hygiene should be performed before putting on personal protective equipment and after removing them [353,358,359,360,361]. To perform hand hygiene, nails should be kept natural (without nail polish, artificial fingernails or extenders) and short (≤0.5 cm). Wearing watches, rings or other jewelry is discouraged, and long-sleeves should be avoided [360]. When hands are not visibly dirty or soiled, the preferred method is to use an alcohol-based hand rub for 20−30 s until they are dry [358,359,360]. Virucidal activity of hand rub agents is tested by EN 14476 (European Committee for Standardization standards) or by ASTM E1838 (American Society for Testing and Materials standards). When hands are visibly dirty or soiled with blood or other body fluids, hands must be washed with plain soap and water for 40−60 s [358,359,360].

Q53—How to perform respiratory hygiene?

Controlling the spread of pathogens from the source is key to avoiding any transmission. Standard respiratory hygiene precautions should be applied to every person exhibiting respiratory symptoms (coughing or sneezing) [358]. Respiratory hygiene precautions are taken during influenza and SARS-CoV epidemics. They are as follows: cover nose and mouth with a disposable/single-used tissue or bent elbow when coughing or sneezing, discard used tissues and masks, and perform hand hygiene after any contact with respiratory secretions or objects potentially contaminated with respiratory secretions [352,358,362]. During COVID-19 outbreak, patients and visitors should wear a medical or cloth mask in the dental facility to prevent the spread of respiratory secretions due to potential asymptomatic and pre-symptomatic transmission [351,353]. Patients should be provided with hand hygiene means, paper tissues and masks in common areas (i.e., reception area and waiting room) [351,352,353,358,363].

### 10.4. Implementing Additional Precautions in COVID-19 Context

Q54—What are additional precautions in COVID-19 context?

Additional precautions are supplementary infection prevention and control measures required by dental staff members to protect themselves and prevent transmission of pathogens like SARS-CoV-2 [363,364]. They include contact, droplet and airborne precautions [362]. During the COVID-19 outbreak, spatial distancing of at least 1–1.5 m should always be maintained between patients [350,351,352,353,363]. It should be also maintained between dental staff members when they need to be unmasked (when eating and drinking) [351]. It can be only broken by dental staff members during a patient’s dental treatment. In addition, use of physical barriers such as glass or plastic panels as protection against respiratory droplets can reduce dental staff members’ exposure to SARS-CoV-2, especially in the reception area [350,351,352,363]. It does not exempt patients and dental staff members from respecting spatial distancing and the use of masks [350].

Q55—How to implement contact and droplet precautions in COVID-19 context?

SARS-CoV-2 is mainly transmitted through respiratory droplets (>5 µm in diameter) and contact routes (see Q32 and Q33). Droplet transmission occurs when a person is in close contact (within 1 m) of infected people. Their mucosae (mouth, nose, eyes) are therefore exposed to infectious respiratory droplets. Transmission can also occur through direct contact with infected people and indirect contact with surfaces (fomites) in the immediate environment or with medical devices previously used on an infected person [352]. Therefore, contact and droplet precautions should be implemented by dental staff caring for each suspected/confirmed COVID-19 patient [349]. They comprise the use of appropriate personal protective equipment (PPE): medical mask, eye protection, non-sterile long-sleeved gown, and medical gloves (see Section 10.5) [352,365]. PPE must fulfil quality standards (European Committee for Standardization [CEN] or American Society for Testing and Materials [ASTM] standards for instance) [354]. A new set of PPE is needed when providing care to a different patient. Dental staff members should refrain from touching their eyes, nose or mouth with potentially contaminated gloved or bare hands [352].

Q56—How to implement airborne precautions in COVID-19 context?

Airborne transmission refers to the presence of droplet nuclei (<5 μm in diameter) which can remain in the air for longer periods of time and can be transmitted to others for distances greater than 1m (see Q32). Airborne transmission of SARS-CoV-2 is possible in settings where aerosol-generating procedures (AGPs) are performed [352]. During the COVID-19 outbreak, airborne precautions should be applied by dental staff for each AGP [350] (e.g., use of high-speed dental turbine and handpiece, air/water syringe, ultrasonic scaler, air polishing, and air abrasion) [351]. They rely on the use of appropriate personal protective equipment: respirator, eye protection, non-sterile long-sleeved gown, and medical gloves. If gowns are not fluid resistant, dental staff members should use an additional water-resistant apron. In addition, the dental treatment room should be ventilated [352].

Q57—When discharging patients from additional precautions?

To relieve patients from isolation, negative rRT-PCR tests are not required [366]. Indeed, the detection of viral RNA does not necessarily mean that a person is contagious. The duration of rRT-PCR positivity generally appears to be 1-2 weeks for asymptomatic patients, and up to 3 weeks or more for symptomatic patients [349].

Criteria for releasing COVID-19 patients from isolation are:

- For symptomatic patients: at least 10 days after symptoms onset (14 to 20 days for severe COVID-19, and 20 days for immunocompromised patients) with an additional 24 to 72 h without fever associated with improvement of respiratory symptoms.
- For asymptomatic cases: 10 days after positive SARS-CoV-2 test [366,367,368].

### 10.5. Using Personal Protective Equipment

Q58—Why using personal protective equipment in COVID-19 context?

Appropriate use of personal protective equipment aims to reduce, but not eliminate, the risks of transmission of respiratory pathogens to dental staff [362].

Q59—How to use gloves in dental facility?

According to standard precautions, medical gloves are indicated in all clinical situations at risk of contact with blood, body fluids, secretions, excretions and items visibly soiled by body fluids, and in cases of contact with mucosae and non-intact skin of patients [359,360,369]. In addition, they are indicated for handling/cleaning instruments, handling waste and cleaning environmental surfaces in the dental facility [359,360]. Their use does not replace the need for proper hand hygiene [359,364]. It is recommended to change them between each patient, and to perform hand hygiene immediately after their removal [358]. Washing or decontaminating gloved hands is strictly prohibited [360,369]. The double gloving is not recommended for COVID-19 patients [363]. Gloves should be removed as soon as they are damaged (or non-integrity suspected). They should also be removed as soon as dental treatment has been completed, and when there is an indication for hand hygiene [356,360].

Q60—Which mask for which situation in dental facility?

Masks are indicated for the protection of healthy people. Wearing a mask allows to protect oneself in case of contact with a COVID-19 patient, and prevents onward transmission of the virus when used by a COVID-19 patient [365]. 

For the general population, the cloth mask is recommended as an alternative to the medical mask during COVID-19 outbreak in public places where there is community transmission and where other prevention measures, such as physical distancing, are not possible [349,365]. Patients and visitors should wear their own cloth mask upon arrival and throughout their stay in the dental facility. Patients may remove them in the dental treatment room, but they must put it back on at the end of dental treatment [351]. For dental staff, the use of cloth masks as an alternative to medical masks is not considered appropriate [363,365] because cloth masks are not personal protective equipment [351]. In addition, cloth masks are not fluid-resistant and thus may retain moisture, become contaminated, and act as a potential source of infection [363].

Medical masks—also known as surgical masks—are indicated for dental staff member and at-risk individuals [365]. Continued use of a medical mask by dental staff members is recommended during all routine activities throughout the entire shift [349,351,353]. Dental staff members caring for COVID-19 patients without aerosol-generating procedures (AGPs) may wear a medical mask. Medical masks should be type IIR (EN 14683 [European Committee for Standardization standards] or tested by ASTM F2100 [American Society for Testing and Materials standards]) [365].

Particulate respirators—also known as filtering facepiece respirator—offer greater filtration capacity. Whereas medical masks filter 3 µm droplets, respirators filter out 0.075 µm solid particles [365]. Thus, medical masks do not offer adequate respiratory protection against aerosols (droplet nuclei), especially due to leaks around the edge of the mask when the user inhales [362]. Use of a respirator is required in dental treatment room where AGPs are performed, especially for COVID-19 patients [351,353,365,370]. In addition, according to ECDC and CDC, respirators are indicated when managing a suspected/confirmed COVID-19 patient (with or without AGPs) [351,353,370]. Respirators should be FFP2 or FFP3 (EN 149; European standards), N95 (NIOSH-42CFR84.181; US standards), or KN95 (GB 2626-2006; Chinese standard) [365]. Moreover, respirators with exhalation valves should not be used during surgical procedures as they allow unfiltered exhaled breath to escape [351,352].

To date, WHO, ECDC and CDC recommendations did not change regarding mask use despite the emergence of new SARS-CoV-2 variants, which have led to increased transmissibility [371,372,373]. However, some countries no longer accept cloth mask for the general population in certain places (e.g., hospitals, public transportation) and extend the use of respirators.

Q61—How to use a mask/respirator?

Correct use of mask/respirator consists in performing hand hygiene before putting on the mask, then placing the mask/respirator on carefully, ensuring it covers the mouth and nose, adjusting it to the nose bridge, and tying it securely to minimize any gaps between the face and the mask/respirator, and finally avoiding touching the mask/respirator while wearing it [365]. Regarding respirator, an initial fit testing is needed before use [352,370]. If the dental staff member has a beard, this may prevent proper fit of the respirator [352]. Mask/respirator should be removed if it is wet, soiled or damaged, if it is exposed to splashes, if it is touched or displaced from face for any reason [363,365]. The use of the same medical mask/respirator by a dental staff member between a confirmed/suspected COVID-19 patient and a patient who does not have COVID-19 is not recommended due to the risk of transmission [363]. Mask/respirator should be removed without touching their front, then a hand hygiene should be performed [365].

Q62—Can dental staff members extend the period of use of their masks/respirators?

Medical mask and respirator are single-used personal protective equipment (PPE). They should ideally be changed after each patient [351,362]. However, during COVID-19 outbreak, which created severe shortages of PPE, medical masks and respirator could be used by dental staff without removing them for up to 6h and 4h, respectively [363,364]. However, wearing medical mask during a prolonged period increases the risk of contamination of the mask/respirator with SARS-CoV-2 and other pathogens. There is a risk that dental staff members will contaminate their hand by touching the front of the mask/respirator. If it is touched/adjusted, hand hygiene must be performed immediately [363]. The risk of contamination can be reduced by wearing a face shield over the mask [356]. Finally, wearing the same medical mask/respirator is only allowed to treat several patients who have the same COVID-19 status [356,364]. Methods of reprocessing medical mask/respirator—by disinfection or sterilization—are neither well established nor standardized. No evidence is available to date on the reprocessing of medical mask/respirator [363].

Q63—How to use eye protection?

Eye protection—such as goggles and face shield—are indicated to reduce the risk of droplets transmission and splashes to the ocular mucosa [365,370]. Face shield covers and protects the entire face from splashes, including the side of the face and the chin [363]. Conventional eye glasses should not be used as eye protection [362]. During COVID-19 outbreak, dental staff should wear eye protection associated with their medical mask/respirator during all patient care [351]. Immediately after removal, goggles and face shield should be decontaminated, and hand hygiene should be performed [363].

Q64—How to use gowns?

According to the additional precautions, a long-sleeved water-resistant non-sterile gown is indicated to protect skin and prevent soiling of work clothes during treatment and activities that may generate splashes of blood or body fluids, and during aerosol-generating procedures (AGPs) [356,358,370]. When used, gowns should always be changed after each patient contact [356]. Immediately after removal, single-use gowns should be discarded and hand hygiene is required [358]. Cloth gowns can be decontaminated for reprocessing by machine washing them at high temperature (60–90 °C) and laundry detergent [363]. If gowns are not water-resistant, dental staff should use an additional disposable water-resistant apron over the gown [352,370]. Water-resistant plastic aprons should not be used alone when performing AGPs on COVID-19 patient [363].

Q65—In which order should personal protective equipment be put on and removed during dental treatments?

Before dental cares, CDC and ECDC suggest the following sequence to put on personal protective equipment (PPE): (1) perform hand hygiene, (2) put on a clean gown or apron, (3) put on a medical mask/respirator, (4) put on eye protection, and (5) put on clean gloves [351,370]. After completion of dental cares, CDC suggests the following sequence to remove PPE: (1) remove gloves, (2) remove gown or apron, (3) perform hand hygiene, (4) remove eye protection, (5) remove and discard surgical mask/respirator, and (6) perform hand hygiene [351].

### 10.6. Environmental Cleaning and Disinfection, and Waste Management

Q66—How to perform environmental cleaning and disinfection in COVID-19 context?

Procedures for cleaning and disinfecting the dental environment aim to reduce any role fomites may play in the transmission of SARS-CoV-2. The SARS-CoV-2 virus remained viable for up to a few days on surfaces, but it is an enveloped virus with a fragile outer lipid envelope that makes it sensitive to disinfectants [374]. Materials, objects, and devices should be stored in a way that facilitates environmental cleaning and disinfection [350]. In the waiting room, toys, magazines, books or other non-essential items that patients may touch should be removed [350,351]. All surfaces in dental facility should be regularly cleaned and disinfected, especially high-touch surfaces, and whenever they are visibly soiled or contaminated with body fluids [352,363]. In common areas, high-touch surfaces require regular cleaning at least twice a day. In dental treatment rooms, high-touch surfaces should be disinfected after each patient visit [350,374] and terminal cleaning is required for low-touch surfaces, high-touch surfaces and floors at least once a day [374].

After ventilation, surfaces should be thoroughly cleaned using a detergent-disinfectant product effective against viruses following the manufacturer’s instructions [350,351,375]. Virucidal activity of disinfectants is tested by EN 14476 (European Committee for Standardization standards) or by ASTM E1053 (American Society for Testing and Materials standards). Cleaning should progress systematically to avoid missing areas, from the least soiled (cleanest) to the most soiled (dirtiest), and from higher to lower levels [374]. Cleaners should wear adequate personal protective equipment: water-resistant apron (or a long-sleeves water-resistant gown after a suspected/infected COVID-19 patient), gloves, medical mask (or respirator in a room were aerosol-generating procedures have been performed) and eye protection [374,375].

No-touch disinfection technology, such as UV irradiation or vaporized hydrogen peroxide, can complement but not replace the first manual cleaning of environmental surfaces that are required to remove organic material [374]. The effectiveness of alternative disinfection methods (e.g., ultrasonic waves, UV irradiation, and blue LED light) against SARS-CoV-2 are not known [351].

Q67—Should sterilization protocols be adapted for SARS-CoV-2?

Dental staff should perform routine cleaning, disinfection, and sterilization protocols of medical devices [351].

Q68—How to laundry work clothes?

To decontaminate work clothes, machine wash at high temperature (60–90 °C) for at least 30 min and the use of laundry detergent is recommended [361]. If a hot-water cycle cannot be used, bleach or other laundry products for decontamination of textiles should be added to the wash cycle [375].

Q69—How to manage waste?

Healthcare waste generated during the care of suspected/confirmed COVID-19 patients are considered as infectious clinical waste and should be collected safely in clearly marked lined containers and sharp safe boxes [352,356,361,375]. Waste are disposed at least once a day [374]. Waste generated in the waiting room can be classified as non-hazardous and should be disposed of in sturdy black bags before being collected by municipal waste management services [361].

### 10.7. Limiting Indoor Air Contamination during the COVID-19 Outbreak

Q70—How to minimize indoor air contamination during dental cares?

For suspected/confirmed COVID-19 patients, aerosol-generating procedures (AGPs) should be avoided as much as possible. When the AGP is required for dental treatment and cannot be postponed, the risk can be minimized by performing a preprocedural mouth rinse, applying rubber dam isolation, using evacuation aspirators/suction and practicing four-handed dentistry [350,351]. If an AGP was performed, the dental treatment room needs to be naturally or mechanically ventilated before admitting a new patient [350].

Q71—How to ventilate the dental treatment room?

Adequate ventilation with fresh and clean outdoor air can play an important role to prevent the spread of airborne infections by reducing the concentration of infectious respiratory aerosols in indoor air. There are three methods for ventilating: natural (window), mechanical, and mixed-mode ventilation [352,362,376]. In dental treatment rooms, a minimum of 6 (ideally 12) air changes per hour is recommended by CDC and ECDC [350,351]. WHO recommends an average natural ventilation rate ≥ 60 L/s/patient or ≥ 12 air changes per hour for mechanical ventilation in an outpatient room with airborne precautions [376].

Q72—Are air cleaners helpful to decontaminate the indoor air?

Air cleaners using a high-efficiency particulate air (HEPA) filter may be effective in reducing the concentrations of infectious aerosols for dental offices without adequate natural or mechanical ventilation [352,375,377]. However, the evidence for the effectiveness of HEPA filters in preventing coronavirus transmission is currently limited [352,356]. If used, the CDC recommends placing the HEPA unit near the dental chair—but not between a dental staff member and the patient’s mouth—and it should not draw air into or through the breathing zone of the dental staff [351].

Air cleaners using ultraviolet germicidal irradiation, air ionizers using negative ion and ozone generators have been proposed in addition to ventilation [351,354,376]. However, the evidence on their effectiveness is currently limited and they are potentially hazardous to human health [377].

## 11. Conclusions

In the course of twelve months, this new virus will have devasted the world order and challenged our medical practices. Starting from virtually nothing, knowledge about SARS-CoV-2 is enriching daily, often overthrowing the approaches of the day before. 

The answers to these 72 questions were submitted to give the reader a current state of science in this field. With this review, we have given a broad overview about SARS-CoV-2, in particular its behavior and transmission abilities, and COVID-19 on a global scale. This manuscript briefly explains how the patients respond to the infection, the symptoms with a focus on oral manifestations, the risk factors and comorbidities, but also the strategies that have been developed to counter the viral spread. As dental professionals are particularly exposed to COVID-19, due to their practice in a potentially contaminated environment, one of the objectives of this review was to inform them of the risks of being infected and therefore transmitting the virus. Thus, we focused on the role played by the oral route in the infection and transmission of SARS-CoV-2, leading to recommendations related to infection prevention and control in dental facilities based on guideline from national and international health agencies.

Finally, the only attitude to be held is to consider each patient as a potential carrier of the SARS-CoV-2 or of another infectious agent. From these data, the reader should be able to master the further knowledge and fully play his role as health actor with his patients. With the difficulties to provide dental healthcare in these specific conditions and the requirement to mobilize all the sanitary resources, it is essential to rethink the role of dentists and to give them a greater space in an integrated medical model.

## Figures and Tables

**Table 1:** Chemical drugs targeting the different steps of the virus life cycle.

Antiviral molecule	Initial Use	Target in the Viral Cycle		References
Losartan	ACE2 antagonist	ACE2 receptor: protein S binding		[316]
Camostat mesylate	TMPRSS2 protease inhibitor, recommended for the treatment of chronic pancreatitis	Protease TMPRSS2: cleavage of the S protein and release of the fusion peptide		[50]
Nafamostat	Anticoagulant, targets Factor Xa and Thrombin			[317,318]
Umifenovir	Antiviral, fusion inhibitor used against Influenzaviruses A and B	pH of endosomal compartments: fusion of viral and cellular membranes		[319,320]
Chloroquine, Hydroxychloroquine	Anti-malaria, used in the treatment of autoimmune diseases			[318,319]
Lopinavir	Antiretroviral, HIV-1 protease inhibitor	Viral protease: maturation of the viral replication/transcription complex		[321,322,323]
Ritonavir	Antiretroviral, HIV-1 protease inhibitor			[322,323]
Darunavir	Antiretroviral, HIV-1 protease inhibitor			[319]
Danoprevir	Antiviral, used for VHC treatment			[324,325]
Remdesivir	Antiviral, developed against Ebolaviruses	RNA dependent RNA polymerase (RdRp)	Nucleoside analog (adenine)	[318,319]
Favipiravir	Antiviral, approved for Influenzaviruses treatment		Nucleoside analog (guanine)	[318,319,326]
Ribavirin	Antiviral, used for hepatitis C (HCV) treatment		Nucleoside analog (guanine)	[318,327]
Clevudine	Antiviral, used for hepatitis B (HBV) treatment		Nucleoside analog (pyrimidine)	[328]
Triazavirin	Antiviral, developed for Influenzaviruses treatment		Non-nucleoside inhibitor	[329]
Sofobuvir	Antiviral, used for HCV treatment		Nucleoside analog (pyrimidine)	[327,330]
Galidesivir	Antiviral, developed against HCV, used for Ebolavirus treatment		Nucleoside analog (adenine)	[330]
Azvudine	Antiviral, developed against HCV, tested against HIV-1		Nucleoside analog (cytidine),	[331]
Nitazoxanide	Antiparasitic, used to treat cryptosporidiosis and giardiasis, broad spectrum antiviral	Blocks the maturation of the viral nucleocapsid		[332,333]
